# The caval index: an adequate non-invasive ultrasound parameter to predict fluid responsiveness in the emergency department?

**DOI:** 10.1186/1471-2253-14-114

**Published:** 2014-12-12

**Authors:** Silke de Valk, Tycho Joan Olgers, Mirjam Holman, Farouq Ismael, Jack Johannes Maria Ligtenberg, Jan Cornelis ter Maaten

**Affiliations:** Emergency Department, Department of Internal Medicine, University Medical Center Groningen, UMCG, Hanzeplein 1, 9700 RB Groningen, The Netherlands; Emergency Department, University Medical Center Groningen, UMCG, Hanzeplein 1, 9700 RB Groningen, The Netherlands; Department of Critical Care, University Medical Center Groningen, UMCG, Hanzeplein 1, 9700 RB Groningen, The Netherlands

**Keywords:** Inferior vena cava, Fluid responsiveness, Ultrasound, Shock, Emergency department

## Abstract

**Background:**

Fluid therapy is the first important step in patients with signs of shock but assessment of the volume status is difficult and invasive measurements are not readily available in the emergency department. We have investigated whether the respiratory variation in diameter of the inferior vena cava is a reliable parameter to predict fluid responsiveness in spontaneous breathing emergency department patients with signs of shock.

**Methods:**

All patients admitted to the emergency department during a 15 week period were screened for signs of shock. If the attending physician planned to give a fluid challenge, the caval index was determined by transabdominal ultrasonography in supine position. Immediately afterwards 500 ml NaCl 0.9% was administered in 15 minutes and the clinical response was observed. An adequate response was defined as an increase in systolic blood pressure of at least 10 mm Hg. Based on this definition patients were divided into responders and non-responders.

**Results:**

After selection a total number of 45 patients was included. A low caval index (< 36.5%) in patients with signs of shock reliably predicted the absence of an adequate response to fluid therapy (negative predictive value 92%). The positive predictive value of a high caval index was much lower (48%) despite the fact that responders had a significantly higher pre-infusion caval index than non-responders (48.7% vs 31.8%, p 0.014).

**Conclusions:**

In spontaneously breathing patients with signs of shock in the emergency department, a high caval index (>36.5%) does not reliably predict fluid responsiveness in our study, while a low caval index (<36.5%) makes fluid responsiveness unlikely. An explanation for the absence of a blood pressure response in the group of patients with a low high caval index might be that these patients represent a group requiring more volume therapy than 500 ml.

## Background

Fluid therapy is considered to be the first step in the resuscitation of hemodynamically unstable patients with signs of shock in the emergency department (ED) [1]. Although it is almost daily practice, making a quick assessment of volume status remains difficult [2]. Uncorrected hypovolemia as well as excess fluid resuscitation are associated with complications [3, 4]. During the initial resuscitation of hemodynamically unstable patients in the ED invasive monitoring is not readily available. A non-invasive parameter to predict fluid responsiveness would be of great value.

The diameter of the inferior vena cava (IVC) increases when the total blood volume increases and decreases when the total blood volume decreases. The diameter also varies during the respiratory cycle due to the changes in intrathoracic pressure during inspiration and expiration [5]. This variation can be expressed as the caval index (difference between expiratory IVC diameter and inspiratory IVC diameter divided by the expiratory IVC diameter, multiplied by 100%).

Several studies have investigated the use of IVC measurements as a marker of volume status.

A caval index greater than or equal to 50% is strongly associated with a low central venous pressure of 8 mmHg or less in intubated and non-intubated patients in the emergency department [6]. There is also evidence for the use of IVC measurements as a marker of volume status in patients who receive ultrafiltration for congestive heart failure as well as during haemodialysis [7, 8]. Several other studies concluded that ultrasound measurements of the respiratory variation in IVC diameter are useful to estimate volume status in intubated and in non-intubated patients in different settings [9–11].

Studies in intensive care unit patients showed that measurements of the respiratory variation in IVC diameter can be used to predict fluid responsiveness in mechanically ventilated patients [12–15]. Research on the accuracy and feasibility of the caval index to predict fluid responsiveness in the emergency department has not yet been performed.

The aim of this study is to investigate the caval index as a non-invasive parameter to predict fluid responsiveness in non-intubated patients with signs of shock in the ED. To our knowledge this is the first study to address this question in the ED.

## Methods

### Design

The study is a prospective, cross-sectional observational study.

During 15 weeks all patients admitted for the internist or the emergency physician to the ED of the University Medical Center Groningen between 09.00 am and 05.00 pm were screened for signs of shock. Signs of shock were defined as: systolic blood pressure < 90 mm Hg or a decrease ≥ 40 mm Hg in patients with known hypertension, heart rate > 100 beats/minute, capillary refill time > 2 seconds, or lactate concentration > 2 mmol/l. The blood pressure was measured by non-invasive cuff. Patients with one or more signs of shock were included in the study when the attending physician decided to start with intravenous fluid resuscitation of at least 500 ml NaCl 0.9%.

Exclusion criteria were documented ascites, peritoneal dialysis, history of liver transplantation, pregnancy, Kussmaul breathing, the inability of the patient to maintain the supine position for several minutes, the inability to obtain adequate ultrasonographic measurements, and hemodynamic or respiratory instability requiring intubation.

### Protocol

Patient characteristics and vital parameters (heart rate, systolic and diastolic blood pressure, mean arterial pressure, capillary refill time, oxygen saturation, respiratory rate and lactate concentration (arterial or venous sample)) were registered at baseline. Before any fluid therapy was given IVC diameters were measured in supine position; the maximal diameter was measured during expiration and the minimal diameter during inspiration. Subsequently a fluid challenge (500 ml NaCl 0.9%) was given in 15 minutes. Vital parameters were registered 15 minutes later after the initial fluid challenge, and after 60 minutes.

In the absence of invasive hemodynamic measurements an increase in systolic blood pressure is a reasonable parameter in the determination of fluid responsiveness. An adequate response to fluid therapy was defined as an increase in systolic blood pressure of at least 10 mm Hg after a fluid challenge of 500 ml NaCl 0,9% in 15 minutes. This cut-off point is based on the change in vital parameters found in several studies investigating fluid responsiveness in different settings using a fluid challenge [13, 16–19]. Based on this definition patients were divided into two groups; one group of responders and one group of non-responders.

Patients were assessed for the presence of sepsis. Sepsis was defined as the presence of two or more systemic inflammatory response syndrome(SIRS)-criteria, most likely caused by an infection [20].

Informed consent was obtained from every patient. The study was approved by the Medical Ethical committee of the University Medical Center Groningen.

### IVC sonography

The IVC was examined from a subcostal view in a longitudinal section. The maximal and minimal diameter were measured during a normal respiratory cycle, no breathing instructions were given. The IVC was examined where its vessel walls were visualized best, preferably no further than 3 cm caudal to the junction of the right atrium [12, 13, 21–24]. Measurements were taken with the abdominal probe (2-6 MHz) from the ultrasound machine z.one ultra-convertible ultrasound system [Zonare, Mountain View, California]. B-mode was used for orientation, then M-mode was used to get a time-motion record of the IVC. The diameters were measured in M-mode, all measurements were stored for later review.

To avoid the limitation of differing skills in IVC measurement, all measurements were performed by one investigator (S.d.V.). In accordance with the ‘Emergency Ultrasound Guidelines’ from ‘the American College of Emergency Physicians (ACEP)’ this investigator completed a training of 50 supervised IVC evaluations and measurements [25, 26]. All videos were reviewed by an independent expert (F.I.).

### Statistical analysis

The statistical analysis was performed with SPSS PASW-18. Baseline characteristics in responders and non-responders were compared using the Mann Whitney U test, and for nominal variables using the Fisher exact test. The Wilcoxon signed rank test was used to compare paired values before and after fluid therapy. Linear correlation between the change in systolic blood pressure and the caval index was tested using the Spearman Rank method. A receiver operating characteristic (ROC) curve was plotted to determine the threshold value of the caval index which provided the prediction of the response to fluid therapy with the best sensitivity and specificity. A P-value less than 0.05 was considered statistically significant.

## Results

### Inclusion

933 patients were screened for signs of shock in a 15-week period. In case of shock the investigator tried to obtain adequate ultrasonographic IVC diameter measurements. Patients with inadequate measurements (IVC not visible or measurements assessed as inadequate by the independent reviewer) were excluded. 52 patients met the inclusion criteria, 7 had inadequate measurements, so the study population contained 45 patients.

Most patients in the study population were admitted with the diagnosis sepsis (n = 23, most common focus lungs or urinary tract) or dehydration (n = 14).

### Responders versus non-responders

Based on the increase in systolic blood pressure in response to a fluid challenge of 500 ml NaCl 0.9% patients were classified as responders and non-responders. Response was defined as an increase in systolic blood pressure of at least 10 mm Hg. Table 1 shows the baseline characteristic of both groups. The systolic blood pressure is significantly higher in non-responders, the other vital parameters do not significantly differ between the two groups. However the pre-infusion caval index is significantly higher in responders.

**Table 1:** **Baseline characteristics of the study population**

Baseline characteristics	Total (n = 45)	Responders (n = 12)	Non-responders (n = 33)	p
**Male (%)**	25 (55.6%)	8 (66.7%)	17 (51.5%)	0.502
**Age (years)**	57,4 (±16.1)	56,3 (±15.0)	57,8 (±16.7)	0.626
**Systolic blood pressure (mm Hg)**	108,5 (±21.4)	97,1 (±17.8)	112,6 (±21.4)	**0.049**
**Diastolic blood pressure (mm Hg)**	63,6 (±13.9)	62,7 (±13.3)	63,9 (±14.3)	0.867
**Mean arterial pressure (mm Hg)**	73,8 (±14.2)	70,8 (±14.7)	74,9 (±14.1)	0.464
**Heart rate (beats/min)**	111,4 (±19.9)	108,8 (±19.2)	112,3 (±20.3)	0.598
**Capillary refill time (seconds)**	3,33 (±1.2)	3,25 (±1.2)	3,35 (±1.2)	0.958
**Lactate(mmol/l)**	1,9 (±1.1)	2,3 (±1.6)	1,7 (±0.8)	0.355
**Respiratory rate (breaths/min)**	22,3 (±5.4)	22,7 (±6.6)	22,1 (±5.0)	0.560
**Oxygen saturation (%)**	97,9 (±2.1)	98,1 (±2.3)	97,8 (±2.1)	0.674
**Temperature (°C)**	37,7 (±1.3)	37,4 (±1.3)	37,7 (±1.3)	0.528
**History of cardiac disease (%)**	7 (15.6%)	1 (8.3%)	6 (18.2%)	0.655
**Caval index (%)**	36,3 (±19.7)	48,7 (±20.2)	31,8 (±17.8)	**0.014**

### Effect of fluid therapy on vital parameters

15 minutes after the fluid challenge the change in vital parameters (apart from the blood pressure on which the division in responders and non-responders is based) is not significantly different in responders compared to non-responders.

The fluid volume administered in the first 15 minutes was standardized as 500 ml NaCl 0.9%, after 15 minutes the amount of fluid administered depended on the attending physician. The mean amount of fluid administered in 60 minutes for responders was 1209.1 (±321.6) ml and for non-responders 958.6 (±401.8) ml, with a wide individual range from 500 ml to 2300 ml. After 60 minutes the change in vital parameters was not significantly different in responders compared to non-responders.

### Caval index and change in systolic blood pressure

**Figure 1:**
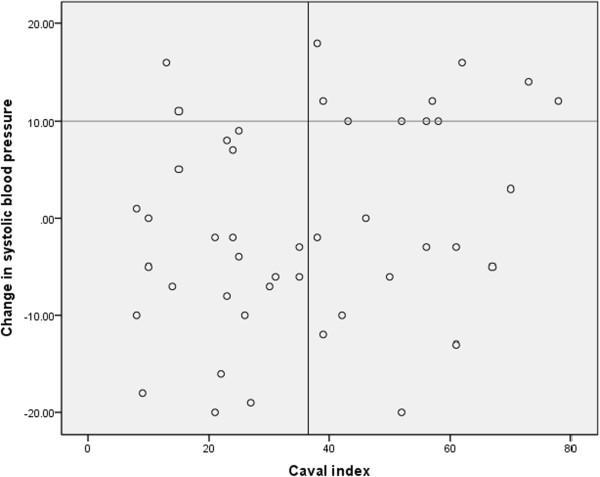
**Relationship between the caval index and the change in systolic blood pressure after a fluid challenge.** The horizontal line divides the study population in a group of responders and a group of non-responders. The vertical line represents the optimal threshold value for the caval index. Test characteristics: sensitivity 83%, specificity 67%, positive predictive value 48%, negative predictive value 92%.

**Figure 2:**
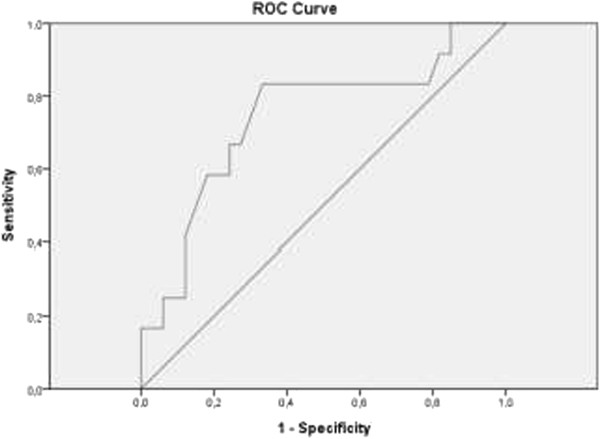
**Receiver operating characteristic (ROC) curve analysis of the caval index as predictor of fluid responsiveness.** Area under the curve 0.741. Optimal threshold value is 36.5% with 83% sensitivity and 67% specificity.

Figure 1 shows there is only a weak relationship between the pre-infusion caval index and the change in systolic blood pressure after a fluid challenge (r = 0.259, p = 0.086). A Receiver Operating Characteristic (ROC) curve shows in this study population an optimal threshold value for the caval index to predict fluid responsiveness of 36.5% (Figure 2). With this threshold value fluid responsiveness (defined as an increase in systolic blood pressure) is predicted with a sensitivity of 83% and a specificity of 67%.

In the non-responders with a high caval index there is a trend to more diagnosis of sepsis (72.7% en 30.0% respectively, p = 0.086) and a trend towards a higher heart rate after 15 minutes (p = 0.051) compared to the responders with a high caval index. Differences are not statistically significant, probably due to low number of patients in both groups.

## Discussion

### Interpretation of findings

Although a caval index < 36.5% in ED patients with signs of shock was associated with fluid unresponsiveness in our study, we could not reproduce a strong correlation between the respiratory variation in IVC diameter and the response to fluid therapy as was previously found in mechanically ventilated intensive care patients.

The pre-infusion caval index in our study population is significantly higher in responders compared to non-responders, but we found only a weak correlation between the caval index and the increase in systolic blood pressure. This is caused by a relatively large group of non-responders with a high caval index but no increase in systolic blood pressure in response to a 500 ml fluid challenge. These patients are more frequently diagnosed with ‘sepsis’ and had a higher heart rate after 15 minutes compared to the group of responders with a high caval index. These findings might indicate that this group was more hypovolemic than the group responders with a high caval index. Considering the large variation in fluid therapy after 60 minutes it is not possible to make any conclusions about the change in vital parameters after 60 minutes (data not shown).

In this study adequate response to a fluid challenge was defined as an increase in systolic blood pressure of at least 10 mm Hg, but this definition of fluid responsiveness might not be suitable. Patients with severe hypovolemic shock (resulting in a high caval index) probably need more fluid therapy to achieve an increase in systolic blood pressure of 10 mm Hg and may therefore be mistaken for non-responders. A remarkable finding was a decrease in systolic blood pressure after the fluid challenge in a considerable part of the patients in our study population in both groups that can be explained by an initial higher blood pressure due to raised stress response during the first minutes in the emergency department.

Our results are in contrast with the findings of Muller and colleagues. They concluded that a high caval index (>40%) in an intensive care unit patient with spontaneous breathing activity is associated with fluid responsiveness whereas a low caval index (<40%) was inconclusive [27]. We found that a low caval index in a patient with signs of shock in the emergency department is associated with fluid unresponsiveness whereas a high caval index is inconclusive. As Bodson and Vieillard-Baron stated in their commentary on the aforementioned study our results are in line with what one should expect based on physiology and previous studies investigating IVC collapsibility, right atrial pressure and central venous pressure [28]. The explanation for the difference in result is not clear. The difference in study population (ED department patient versus ICU patients) may have influenced the results. Possibly ICU patients already received more fluid therapy during the primary resuscitation which makes them more prone to show response to a fluid challenge, whereas ED patients need more volume therapy than 500 ml to show response. Another important difference between the study of Muller and colleagues and our study is the definition of fluid responsiveness. They used a 15% increase of subaortic velocity time index (VTI) measured by transthoracic echocardiography, where we used an increase in systolic blood pressure of at least 10 mmHg.

### Limitations

A limitation of this study is the small study population. Furthermore, we could not obtain adequate ultrasonographic measurements in 13% of the patients who met the inclusion criteria, which was comparable with 10-15% found in previous studies [6, 13, 21, 29]. The study population contains a selected group of patients; patients were only included in the study when the attending physician decided to treat the signs of shock immediately with a fluid challenge. The patients were not aggressively fluid resuscitated, with 2300 ml being the maximum amount given in 60 minutes. This might be due to the fact that patients with severe shock resulting in hemodynamic or respiratory instability requiring intubation were excluded. Moreover in this small study population there was a large heterogeneity in patients, which might have affected the results.

The caval index is not only influenced by the total blood volume, but also by the abdominal pressure and the compliance of the IVC. In our study the abdominal pressure was not measured. No corrections were made for medications such as analgetics or anxiolytics administered to patients. However none of the patients received vasopressor or inotropic agents during the study period. In patients with known right heart failure, severe pulmonary hypertension, severe tricuspid regurgitation the caval index has to be interpreted with caution as any right atrial overload will tend to result in distention of the IVC.

## Conclusion

This study is the first study in the ED investigating the respiratory variation in IVC diameter (caval index) as a parameter for fluid responsiveness using clinical definitions. We found that a caval index of < 36.5% in a patient with signs of shock predicts the absence of an adequate response to a fluid challenge of 500 ml NaCl 0.9% with a reliability of 92%. Aggressive fluid therapy might not be indicated or even harm these patients. However it is not possible to predict the response to a fluid challenge for a patient with a caval index of > 36.5%. This is reflected by the low positive predictive value (48%) and weak correlation between caval index and fluid responsiveness. An explanation for the absence of a blood pressure response might be that these patients represent a group requiring more volume therapy than 500 ml. In future studies we will investigate this issue.

### Key messages

- This is the first study investigating the use of the caval index for the assessment of fluid responsiveness in the emergency department
- In line with physiological expectations and previous studies, a caval index of < 36.5% in a patient with signs of shock predicts the absence of an adequate response to a fluid challenge of 500 ml NaCl 0.9% with a reliability of 92%.
- A high caval index of > 36.5% has a low positive predictive value for fluid responsiveness after a 500 ml fluid challenge, which might reflect the need for more aggressive fluid resuscitation.

## Abbreviations

UMCG: University Medical Center Groningen
ED: Emergency department
IVC: Inferior vena cava
ICU: Intensive care unit
SIRS: Systemic inflammatory response syndrome
ROC: Receiver operating characteristic
VTI: Velocity time index.
